# Heart Rate Recovery as a Predictor of Long-Term Adverse Events after Negative Exercise Testing in Patients with Chest Pain and Pre-Test Probability of Coronary Artery Disease from 15% to 65%

**DOI:** 10.3390/diagnostics13132229

**Published:** 2023-06-30

**Authors:** Vojislav Giga, Nikola Boskovic, Ana Djordjevic-Dikic, Branko Beleslin, Ivana Nedeljkovic, Goran Stankovic, Milorad Tesic, Ivana Jovanovic, Ivana Paunovic, Srdjan Aleksandric

**Affiliations:** 1 Cardiology Clinic, University Clinical Center of Serbia, 11000 Belgrade, Serbia; belkan87@gmail.com (N.B.); skali.ana7@gmail.com (A.D.-D.); branko.beleslin@gmail.com (B.B.); ivannanedeljkovic@yahoo.com (I.N.); gorastan@gmail.com (G.S.); misa.tesic@gmail.com (M.T.); ivana170679@gmail.com (I.J.); ina.paunovic@gmail.com (I.P.); srdjanaleksandric@gmail.com (S.A.); 2 School of Medicine, University of Belgrade, 11000 Belgrade, Serbia

**Keywords:** chest pain, negative exercise testing, prognosis, predictors, heart rate recovery

## Abstract

Background: The prognosis of patients with chest pain after a negative exercise test is good, but some adverse events occur in this low-risk group. The aim of our study was to identify predictors of long-term adverse events after a negative exercise test in patients with chest pain and a lower intermediate (15–65%) pre-test probability of coronary artery disease (CAD) and to assess the prognostic value of exercise electrocardiography and exercise stress echocardiography in this group of patients. Methods: We identified from our stress test laboratory database 862 patients with chest pain without previously known CAD and with a pre-test probability of CAD ranging from 15 to 65% (mean 41 ± 14%) who underwent exercise testing. Patients were followed for the occurrence of death, non-fatal myocardial infarction (MI) and clinically guided revascularization. Results: During the median follow-up of 94 months, 87 patients (10.1%) had an adverse event (AE). A total of 30 patients died (3.5%), 23 patients suffered non-fatal MI (2.7%) and 34 patients (3.9%) had clinically guided revascularization (20 patients percutaneous and 14 patients surgical revascularizations). Male gender, age, the presence of diabetes and a slow heart rate recovery (HRR) in the first minute after exercise were independently related to the occurrence of AEs. Adverse events occurred in 10.3% of patients who were tested by exercise stress echocardiography and in 10.0% of those who underwent stress electrocardiography (*p* = 0.888). Conclusion: The risk of AEs after negative exercise testing in patients with a pre-test probability of CAD of 15–65% is low. Male patients with a history of diabetes and slow HRR in the first minute after exercise have an increased risk of an adverse outcome.

## 1. Introduction

Exercise testing with or without imaging still has an important role in everyday clinical practice in patients with chest pain both for the diagnosis and risk stratification of coronary artery disease (CAD). It has been demonstrated that the presence of inducible ischemia carries a 5–10-fold increased risk for the occurrence of adverse events (AEs) [1,2,3].

However, in recent years, the number of tests positive for myocardial ischemia is decreasing and is currently relatively low (10–15%) [4]. A large meta-analysis including more than 11,000 patients showed that such a negative exercise test coupled with imaging (myocardial perfusion imaging or exercise stress echocardiography) carries good prognosis during a mean follow-up period of around 3 years [5]. A more recent meta-analysis confirmed previous findings showing an annual event risk of death and non-fatal myocardial infarction (MI) of 0.90% after negative exercise electrocardiography, and of 1.77% after negative exercise stress echocardiography after a median follow-up of around 2 years. The higher event rate in the group tested by exercise stress echocardiography can be attributed to the higher population event risk, which reflects common clinical practice where the patients with a higher probability of CAD are referred to exercise testing coupled with imaging [6]. Both meta-analyses had relatively short follow-up periods, included both patients with suspected and known CAD and were not able to identify predictors of adverse events. Data on long-term follow-up assessing the predictors of adverse outcome in patients without known CAD after a negative exercise test are relatively scarce. Adverse events after a negative exercise test may occur in patients with significant coronary artery stenosis that has not been identified by the test (false negative results). On the other hand, a large multicentric study comparing the prognostic value of coronary computed tomography angiography (CCTA) and functional testing showed that a significant number of adverse cardiac events occurred in patients with subclinical atherosclerosis and a negative exercise test [7] due to the presence of non-significant coronary artery stenosis, which cannot be identified by conventional exercise testing but may be responsible for the occurrence of acute events. These data suggest that some AEs occur in the low-risk group of patients and underscore the clinical need to further refine risk stratification in patients with a negative exercise test. Vulnerable patients prone to AEs, having lipid-rich atherosclerotic plaques with a thin cap, can be reliably identified using intracoronary imaging such as optical coherence tomography during invasive coronary angiography [8,9,10]. However, such an approach is related to radiation exposure and high costs. On the other hand, it has been shown that easily obtainable markers of autonomic nervous system activity, such as chronotropic incompetence [11,12] and heart rate recovery (HRR) after exercise [13,14], may identify patients with a pronounced risk of adverse outcome.

Therefore, the aim of our study was to identify predictors of long-term AEs after negative exercise in patients with chest pain and a lower intermediate (15–65%) pre-test probability of CAD. It was of additional interest to assess the prognostic value of negative exercise electrocardiography and exercise stress echocardiography in this group of patients.

## 2. Materials and Methods

### 2.1. Patient Population

We identified from our stress test laboratory database 1005 patients with chest pain without previous CAD (known significant coronary artery stenosis, previous MI and/or coronary revascularization) and with pre-test probability of CAD ranging from 15 to 65%, based on recommended clinical algorithm [15], who underwent exercise testing, stress electrocardiography or exercise stress echocardiography for the evaluation of chest pain from January 2007 to December 2008. Patients with uninterpretable electrocardiograms (ECGs) (left bundle branch block, Wolf–Parkinson–White syndrome and baseline ST-T abnormalities that preclude ECG interpretation), as well as patients with clinical complaints other than chest pain and those having non-cardiac conditions that affect ability to exercise were not considered eligible.

A detailed interview and clinical examination were performed prior to exercise testing in all patients for the assessment of nature of symptoms (typical vs. atypical chest pain) with the estimation of pre-test probability of CAD as previously described [15]. Diabetes mellitus [16], arterial hypertension [17] and hypercholesterolemia [18] were defined according to standard criteria. In addition, smoking status and family history of premature cardiovascular disease were assessed in all patients.

A total of 120 out of 1005 patients (11.9%) had positive exercise test defined as horizontal or down-sloping ST segment depression at 80 ms after the J point of at least 1 millimetre in at least 3 consecutive beats in 2 contiguous leads in the case of exercise ECG or as the development of new wall motion abnormality in at least 2 adjacent segments of left ventricle in the case of exercise stress echocardiography and were excluded from further analysis. After the exclusion of patients lost to follow-up (23 patients), final study population comprised 862 patients.

Patients were followed by phone contact for the occurrence of death, MI or clinically guided revascularization. To avoid misclassification of the cause of death, overall mortality was considered [19,20]. Myocardial infarction was defined by typical symptoms and electrocardiographic and cardiac enzyme changes and confirmed by discharge summary diagnosis.

### 2.2. Exercise Testing

All patients underwent maximal exercise test on treadmill using standard Bruce protocol. Immediately after exercise, patients lay down in a supine position. The decision to perform exercise ECG or exercise stress echocardiography was left to the discretion of physician performing exercise testing. Data on exercise duration, resting and peak heart rate, as well as on HRR in first minute after exercise, were recorded. Abnormal HRR was defined as ≤18 beats/min, as previously described and validated [21,22]. Chronotropic index, as a measure of chronotropic incompetence, was calculated by a formula [(peak heart rate–resting heart rate)/(220–age–resting heart rate)] and considered abnormal if <0.80 [23]. Blood pressure was monitored at baseline and at each stage of the exercise. Presence of symptoms during testing was assessed. Beta blockers were stopped for 48 h prior to exercise testing in all patients.

Exercise stress echocardiography was performed according to standard procedure. All echocardiographic images were obtained at rest and within 1 min after the peak exercise in recumbent (left lateral decubitus) position and digitally stored for analysis. Regional wall motion was assessed using 17-segment model as recommended [24].

### 2.3. Statistical Analysis

Continuous variables were reported as mean ± SD, and differences were assessed with the unpaired t test or Mann–Whitney U test as appropriate. Normal distribution of all continuous variables was confirmed by Kolmogorov–Smirnov test. Categorical variables were reported as percentages and compared between groups by chi-square test. Univariate analysis was used to evaluate the relation between various clinical and hemodynamic variables during exercise and occurrence of adverse events in the follow-up period. Event-free survival curves for adverse events were estimated by Kaplan–Meier method and compared using the log-rank test. Univariate and multivariate (enter method) Cox proportional hazards models were used to assess predictors of adverse events. A significance of 0.05 was required for a variable to be included into the multivariate model. Hazard ratios with the corresponding 95% confidence intervals were estimated. Statistical significance was defined as *p* ˂ 0.05. Statistical Package for the Social Sciences (SPSS release 25.0, Chicago, IL, USA) was used for the analysis.

## 3. Results

**Figure 1:**
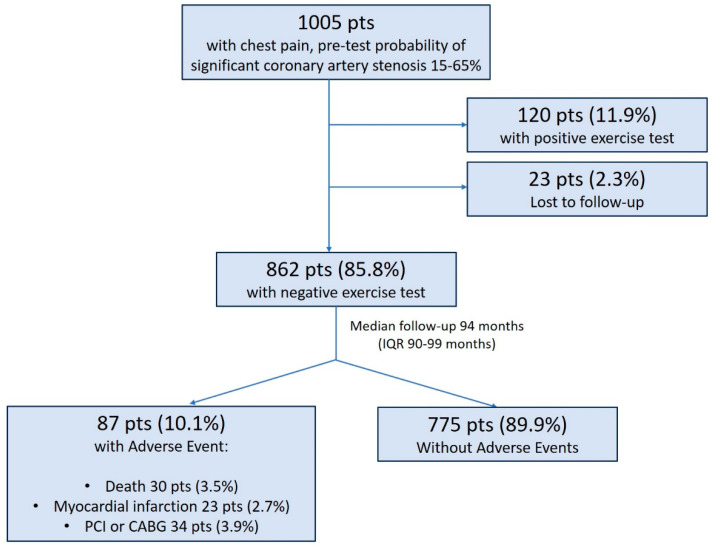
Study flow chart with main outcome results.

The final study population comprised 862 patients (mean age 56 ± 10 years, 42% of male patients). The mean pre-test probability of CAD was 41 ± 14%. A study flow chart with the main outcome results is presented in Figure 1.

Baseline clinical characteristics of the study population and exercise data are summarized in Table 1. Patients were treated with statins (202 (24%)), acetylsalicylic acid (366 (44%)), ACE inhibitors/angiotensin receptor blockers (203 (24.4%)) and calcium channel blockers (181 (21%)).

**Figure 2:**
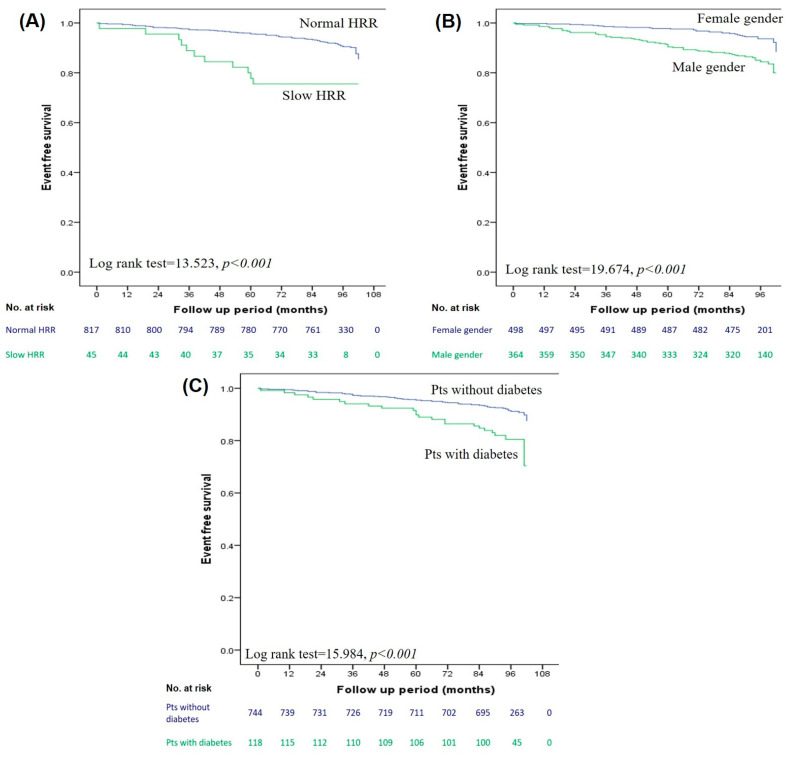
Kaplan–Meier analysis for adverse events according to heart rate recovery (**A**), gender (**B**), diabetes (**C**).

During the median follow-up of 94 months (IQR 90–99 months), 87 patients (10.1%) had an AE. A total of 30 patients died (3.5%), 23 patients suffered non-fatal MI (2.7%) and 34 patients (3.9%) had clinically guided revascularization (20 patients percutaneous and 14 patients surgical revascularizations). The annual rate of AEs was 1.3%. The median time to AE was 60 months (IQR 30–84 months). Patients with AEs were predominantly males, were older and had a significantly higher prevalence of diabetes and typical chest pain. Regarding exercise data, patients with AEs had a higher prevalence of slow HRR and impaired chronotropic index. A comparison of clinical and exercise data between patients with and without AEs is presented in Table 1. Univariate predictors of AEs are summarized in Table 2. Patients with a slow HRR after exercise had a significantly lower event-free survival time in comparison to patients with a preserved HRR (86.7 ± 4.2 vs. 97.9 ± 0.5 months, log-rank test 13.523, *p* < 0.001) (Figure 2A), as well as male patients (94.2 ± 1.1 vs. 99.6 ± 0.5 months for females, log-rank test 19.674, *p* < 0.001) (Figure 2B) and those with diabetes (92.9 ± 2.1 vs. 98.1 ± 0.6 months for non-diabetic, log-rank test 15.984, *p* < 0.001) (Figure 2C). Male gender, age, the presence of diabetes and a slow HRR were independently related to the occurrence of AE (Table 2).

Overall, stress ECG was performed in 541 patients (63%), whereas exercise stress echocardiography was performed in 321 patients (37%). There was no significant difference in patient characteristics and exercise data between patients undergoing stress electrocardiography and those undergoing exercise stress echocardiography except for a somewhat higher pre-test probability of CAD in the latter group (Table 3). The rate of AEs was similar in two groups. Adverse events occurred in 10.3% of patients who were tested by exercise stress echocardiography and in 10.0% of those who underwent stress electrocardiography (*p* = 0.888).

## 4. Discussion

The current study demonstrated that patients with chest pain and a pre-test probability of CAD of 15–65% have a low incidence of adverse events after a normal exercise test during long-term follow-up. The presence of diabetes and an impaired heart rate recovery in the first minute after exercise can further identify those with a poorer outcome.

A negative exercise test is a common finding in everyday clinical practice. The recent meta-analysis revealed a negative finding in 72% of exercise stress echocardiography tests (36 studies, including 28% of patients with known CAD), whereas the negativity rate was 75% for studies using stress electrocardiography (21 studies, including 23% of patients with known CAD) [6]. Similarly, a recent study from high-volume centers reported 80% of negative findings in exercise stress echocardiography after the 2000s in patients without previously known CAD [25]. The rate of the positive exercise test was even lower in our group of patients (11.9%). This difference probably reflects the fact that, in our study, we included only patients with a lower intermediate pre-test probability of CAD. The vast majority of our patients had a negative result in exercise testing, and such a finding carries an excellent prognosis. During a median follow-up of 94 months, 10.1% of patients with a negative test had an AE defined as death, MI or clinically guided revascularization with an annual event rate of 1.3%. When accounted for hard events (death, MI), the annual rate was 0.84%. A large meta-analysis evaluating the prognostic value of negative non-invasive cardiac investigations in patients with suspected or known CAD revealed annualized event rates of cardiac death and MI of 1.77% with exercise stress echocardiography and 0.9% with stress electrocardiography [6]. An adverse event rate, defined as an overall mortality or MI, of >2% was observed in negative exercise stress echocardiography tests after the 2000s in a recent study [25]. A study by Bangalore et al. confirmed that the negative exercise test also has a low hard cardiac event rate (less than 1% per year) in patients stratified according to different pre-test probabilities for the presence of CAD [26]. A significant proportion of AEs in our study were clinically guided revascularizations (percutaneous or surgical), with an annualized rate of 0.46%. In a previously published meta-analysis, the annual rate of unstable angina and revascularization was 0.95% after negative exercise stress echocardiography testing in patients with known or suspected CAD [5]. According to published data, even when both positive and negative test results are considered, the 60-day revascularization rate after an exercise test remains low (2.7%) in patients without previously known CAD [27], whereas the annual rate of revascularization after negative exercise stress echocardiography in patients without known CAD was reported to be around 3% [25]. Moreover, patients with severe to moderate ischemia, confirmed by different non-invasive tests, treated by optimal medical therapy have the same risk of ischemic cardiovascular events or death as those treated by revascularization, so a further decrease in revascularization procedures is expected [28]. A significantly lower revascularization rate after negative exercise testing in the current study confirms the low-risk nature of our study population. In our study, AEs occurred with a median of 60 months after negative exercise testing, with the range from 1 to 87 months. The early occurrence of AEs, especially clinically guided revascularizations, can be attributed to false negative test results. On the other hand, AEs in later stages of follow-up may reflect disease progression even in this group of low-risk patients. In addition, some of the AEs might be caused by the rupture of non-obstructive plaques that cannot be identified by exercise testing.

In this group of patients with negative exercise test results, the risk could be further stratified with the interaction of clinical characteristics (male gender, age, presence of diabetes) and a slow HRR in the first minute after exercise. Recently, the importance of classical risk factors for risk stratification has once more been emphasized in the contemporary patient population with chest pain and normal functional testing [7]. Namely, the addition of the Framingham Risk Score in a large-scale PROMISE trial significantly improved the discriminatory capacity of functional testing, which rendered the comparison to anatomic testing using CCTA non-significant [7]. Similarly, a study by Cortigiani et al. using exercise stress echocardiography for prognostic assessment in 14,140 patients, of whom 2835 were diabetics, showed that the prognosis after negative exercise stress echocardiography is far less benign in diabetic patients than in non-diabetic patients [29]. Our results confirm these findings and demonstrate that the presence of diabetes is a marker of a less favorable prognosis even in low-risk patients, i.e., younger patients without known CAD and without inducible ischemia.

The drop in heart rate after exercise reflects the interplay between sympathetic withdrawal and parasympathetic reactivation [30,31], with the latter one being clinically more important since it has been shown that a reduced vagal activity has an adverse impact on mortality [32]. A slow HRR has been related to an increase in overall mortality [33,34,35] and increased incidence of cardiovascular events [13,14]. The prognostic value of HRR has been demonstrated in the whole spectrum of subjects: in apparently healthy and asymptomatic populations [13,14] and in patients with suspected and known CAD [21,36,37,38]. Our data extend previous knowledge by demonstrating that an impaired HRR, defined as the inability to decrease the heart rate for more than 18 beats in the first minute after exercise, retains the prognostic ability for the occurrence of AEs in the highly selected group of patients with chest pain, a lower intermediate pretest probability of CAD and clearly negative test results. An impaired HRR was associated with a higher incidence of overt and silent myocardial ischemia in long-term follow-up [39], suggesting its relation to CAD progression, a finding that might also explain some AEs that occurred late in our study.

From a pathophysiological point of view, a slow HRR after exercise has been linked to endothelial dysfunction [40], inflammation [41], increased arterial stiffness [42] and insulin resistance [43], factors that may accelerate the progression of atherosclerosis. Moreover, patients with an impaired HRR are more likely to have subclinical atherosclerosis, demonstrated by higher values of the coronary artery calcium score [44]. Additionally, patients with a slow HRR after exercise have impaired fibrinolysis expressed as elevated plasminogen activity inhibitor-1 activity, tissue plasminogen activator antigen and fibrinogen with a pronounced risk of atherothrombosis [45]. A higher incidence of AEs in patients with an impaired HRR observed in our study can be explained, on one hand, by a faster progression of atherosclerosis requiring revascularization. On the other hand, patients with an impaired HRR might have a higher prevalence of subclinical disease, which, together with endothelial dysfunction, inflammation and impaired fibrinolysis, may lead to MI and death even in the absence of obstructive CAD.

The last European Guidelines on chronic ischemic heart disease recommend the use of exercise testing coupled with imaging rather than exercise ECG alone for the detection of CAD [46]. Since other mechanisms than coronary artery stenosis may underly chest pain, multimodality imaging integrating “anatomical” and “functional” information is required to elucidate the exact mechanism of chest pain and myocardial ischemia [47].

In our study, we assessed the prognostic value of exercise electrocardiography and exercise stress echocardiography, two widely available tests in everyday clinical practice. Our data showed a similar rate of AEs in patients undergoing exercise stress electrocardiography and exercise stress echocardiography, similarly to some previously published data [48,49]. However, we were not able to compare the prognostic difference between the two diagnostic methodologies with current retrospective analysis. The decision to perform exercise electrocardiography or exercise stress echocardiography was left to the discretion of the physician performing exercise testing. A higher pre-test probability for the presence of obstructive CAD observed in patients referred to exercise stress echocardiography, although reflecting common clinical practice, might impact our study results. Additionally, the negativity of exercise stress echocardiography was based only on the absence of wall motion abnormalities. There is evidence of the declining prognostic value of a negative exercise stress echocardiography based only on regional wall motion abnormalities in contemporary populations [25]. In our study, we did not use a detailed echocardiography evaluation with the assessment of LV volumes, ejection fraction, LV contractile reserve, coronary flow velocity reserve in the left anterior descending artery and pulmonary congestion by the identification of lung B-lines. This ABCDE stress echo protocol is an effective predictor of survival in patients with chronic coronary syndrome, including those with suspected CAD who do not exhibit wall motion abnormalities during exercise or the pharmacological test [50]. At the time when the study was conducted, we routinely assessed only the wall motion abnormalities, so we could not identify all the patients prone to AEs. Moreover, the analysis of global longitudinal strain (GLS) at rest can better identify the presence of significant coronary artery stenosis, defined as the presence of a luminal narrowing of 50%, than stress vasodilator echocardiography by the analysis of the wall motion score index and coronary flow velocity reserve in the left anterior descending coronary artery in patients with a preserved left ventricular ejection fraction [51]. The number of patients with significant coronary artery stenosis would have increased in our study had we conducted an analysis of GLS during rest. The exclusion of these patients with significant but undiagnosed CAD from further analysis would have increased the prognostic value of negative exercise stress echocardiography in the current study.

Overall, patients with a negative exercise test and a lower intermediate pre-test probability of obstructive CAD have good prognosis. It has to be emphasized that a simple marker such as an impaired HRR has a considerable prognostic impact and, above all, is considerably cost saving, the latter being important nowadays [52,53]. Based on our results, we recommend that data on HRR should be included in every exercise testing report.

### Study Limitations

The first limitation of our study is that it represents a single center experience; however, the data come from a high-volume certified center [54] experienced in performing and interpreting stress ECG and exercise stress echocardiography. The European Society of Cardiology Guidelines on chronic coronary syndrome recommended a new model for the assessment of pre-test probability for the presence of CAD that is mainly based on patients from countries with a low cardiovascular disease risk. However, our patient population comes from a country with a high prevalence of CAD; therefore, we used a previously recommended algorithm for pre-test probability assessment [15]. This tool evaluates the pre-test probability of a coronary artery stenosis of more than 50% during invasive angiography. Nevertheless, in our study, we considered the clinical outcome as the endpoint. These two measurements differ for several reasons. Firstly, the overall death could be unrelated to CAD. Moreover, angina may be caused by a stenosis of less than 50% with a hemodynamic significance or with a non-obstructive mechanism (i.e., angina with non-obstructive coronary arteries), such as microvascular dysfunction [55,56] or vasospasm [57]. In addition, it was highlighted that 6–8% of MIs may develop in the presence of non-significant coronary stenosis [58]. These patients may be identified by invasive coronary angiography or CCTA. However, we did not collect data on coronary angiography if it was not followed by revascularization. Finally, long-term outcomes were evaluated, so even if significant CAD might have been absent at the time of the exercise test, it could have developed during the follow-up period. Coronary revascularization was included as an outcome in the current study. The decision to perform coronary angiography and revascularization was made by referring physicians so we cannot exclude the possibility that some of the revascularizations were performed on intermediate stenosis without proof of ischemia. On the other hand, we were not aware of the data if additional functional testing that led to revascularization was performed during the follow-up period. In addition, we were not aware of the changes in medical therapy or life-style modification during the follow-up period so we cannot exclude the possibility that these changes might affect the outcome of the patients. We analyzed only patients undergoing exercise testing, so our data cannot be extrapolated to patients undergoing pharmacological testing. Therefore, patients unable to exercise, such as the elderly or patients with peripheral artery disease, who are at high risk for CAD, were not analyzed in the current study [59]. In this study, we could only analyze the data obtained on HRR in the first minute after exercise. Although it has been postulated that 2 min HRR might be more sensitive in predicting the risk of cardiovascular events than 1 min HRR [30], the latter has been previously validated in a study using exercise stress echocardiography for the detection of myocardial ischemia with patients in a supine position after exercise [11].

## 5. Conclusions

The risk of adverse events after negative exercise testing in patients with a pre-test probability of CAD of 15–65% is low. However, male patients with a history of diabetes and slow HRR in the first minute after exercise have an increased risk of an adverse outcome and require special clinical attention.

## Figures and Tables

**Table 1:** Baseline clinical characteristics and exercise data.

Variable	All Patients (*n* = 862)	Patients with AE ^a^ (*n* = 87, 10.1%)	Patients without AE ^a^ (*n* = 775, 89.9%)	*p* Value
Male gender	364 (42.2%)	56 (64.4%)	308 (39.7%)	<0.001
Age (years)	56 ± 10	60 ± 10	55 ± 10	<0.001
Hypertension	603 (70%)	67 (77%)	536 (69.2%)	0.130
Hyperlypoproteinemia	488 (56.6%)	50 (57.5%)	438 (56.5%)	0.865
Smoker	326 (37.9%)	38 (44.2%)	288 (37.2%)	0.203
Diabetes	118 (13.7%)	24 (27.6%)	94 (12.1%)	<0.001
Family history of CAD ^b^	509 (59%)	49 (56.3%)	460 (59.4%)	0.585
Typical chest pain	403 (48.4%)	71 (81.6%)	498 (64.3%)	0.001
Duration of the test (minutes)	7.4 ± 2.7	7.0 ± 2.7	7.5 ± 2.8	0.132
Chronotropic index < 0.8	411 (47.7%)	54 (62.1%)	364 (47%)	0.008
Achieved target heart rate	674 (78.2%)	62 (71.3%)	612 (79%)	0.099
Maximum achieved SBP ^c^ (mmHg)	180 ± 21	183 ± 21	179 ± 22	0.126
Maximum achieved DBP ^d^ (mmHg)	100 ± 11	100 ± 11	100 ± 11	0.946
Slow HRR ^e^	45 (5.2%)	11 (12.6%)	34 (4.4%)	0.001

^a^ AEs—adverse events (death + myocardial infarction + CABG + PCI), ^b^ CAD—coronary artery disease, ^c^ SBP—systolic blood pressure, ^d^ DBP—diastolic blood pressure, ^e^ HRR—heart rate recovery.

**Table 2:** Univariate and multivariate Cox proportional hazards analysis for AE ^a^.

Variable	HR ^h^	Univariate Analysis		B	*p* Value	Multivariate Analysis			
		95% CI ^i^				HR ^h^	95% CI ^i^		*p* Value
		Lower Level	Upper Level				Lower Level	Upper Level	
Male gender	2.600	1.676	4.032	0.955	<0.001	2.525	1.441	4.425	0.001
Age (years)	1.045	1.021	1.070	0.044	<0.001	1.042	1.017	1.067	0.001
Hypertension	1.542	0.935	2.543	0.433	0.089				
Hyperlipoproteinemia	1.086	0.698	1.634	0.066	0.762				
Smoking	1.395	0.910	2.139	0.333	0.126				
Diabetes	2.523	1.576	4.038	0.925	<0.001	1.891	1.171	3.055	0.009
Family history of CAD ^b^	0.894	0.585	1.365	−0.113	0.603				
Typical chest pain	2.495	1.449	4.296	0.914	0.001	0.838	0.420	1.670	0.615
SECHO ^c^/SECG ^d^	0.989	0.641	1.525	−0.011	0.959				
Duration of the test (minutes)	0.945	0.875	1.021	−0.056	0.151				
Achieved target heart rate	0.682	0.429	1.086	−0.382	0.107				
Maximum achieved SBP ^e^	1.007	0.997	1.017	0.007	0.157				
Maximum achieved DBP ^f^	1.000	0.982	1.019	0.000	0.976				
Chronotropic index < 0.8	1.765	1.144	2.724	−0.568	0.010	1.493	0.955	2.332	0.079
Slow HRR ^g^	3.084	1.638	5.808	1.126	<0.001	2.024	1.041	3.939	0.038

^a^ AEs—adverse events (death + myocardial infarction + CABG + PCI), ^b^ CAD—coronary artery disease, ^c^ SECHO—stress echocardiography, ^d^ SECG—stress electrocardiography, ^e^ SBP—systolic blood pressure, ^f^ DBP—diastolic blood pressure, ^g^ HRR—heart rate recovery, ^h^ HR—hazard ratio, ^i^ CI—confidence interval.

**Table 3:** Comparison of baseline clinical characteristics, exercise data and outcome in patients tested with exercise stress echocardiography and stress electrocardiography.

Variable	All Patients (*n* = 862)	SECHO ^a^ (*n* = 321, 37.2%)	Stress ECG ^b^ (*n* = 541, 62.8%)	*p* Value
Male gender	364 (42.2%)	137 (42.7%)	227 (42%)	0.836
Age	56 ± 10	56 ± 9	56 ± 10	0.245
Hypertension	603 (70%)	227 (70.7%)	376 (69.5%)	0.707
Hyperlypoproteinemia	488 (56.6%)	190 (59.2%)	298 (55.1%)	0.240
Smoker	326 (37.9%)	111 (34.7%)	215 (39.7%)	0.140
Diabetes	118 (13.7%)	45 (14%)	73 (13.5%)	0.828
Family history of CAD ^c^	509 (59%)	190 (59.2%)	319 (59%)	0.948
Typical chest pain	293 (34%)	119 (37.1%)	174 (32.2%)	0.141
PTP ^d^ (%)	40.7 ± 13.9	42.3 ± 14	39.7 ± 13.8	0.009 ^l^
Duration of the test (minutes)	7.4 ± 2.7	7.4 ± 2.9	7.5 ± 2.7	0.764
Chronotropic index < 0.8	418 (48.5%)	158 (49.2%)	260 (48.1%)	0.761
Achieved target heart rate	674 (78.2%)	255 (79.4%)	419 (77.4%)	0.494
Maximum achieved SBP ^e^ (mmHg)	180 ± 21.1	180 ± 21.2	180.1 ± 21.1	0.814
Maximum achieved DBP ^f^ (mmHg)	100.4 ± 11.3	99.9 ± 12	100.5 ± 10.8	0.509
Slow HRR ^g^	45 (5.2%)	16 (5%)	29 (5.4%)	0.810
AE ^h^	87 (10.1%)	33 (10.3%)	54 (10.0%)	0.888

^a^ SECHO—exercise stress echocardiography, ^b^ ECG—electrocardiogram, ^c^ CAD—coronary artery disease, ^d^ PTP—pre-test probability (Diamond–Forrester), ^e^ SBP—systolic blood pressure, ^f^ DBP—diastolic blood pressure, ^g^ HRR—heart rate recovery, ^h^ AEs—adverse events (death + myocardial infarction + CABG + PCI).

## Data Availability

The raw data supporting the conclusions of this article will be made available by the authors, without undue reservation.
